# Loss of CCDC188 causes male infertility with defects in the sperm head-neck connection in mice

**DOI:** 10.1093/biolre/ioae137

**Published:** 2025-01-14

**Authors:** Yumiao Qiu, Keisuke Shimada, Kaito Yamamoto, Masahito Ikawa

**Affiliations:** 1 Research Institute for Microbial Diseases, Osaka university, Osaka, 565-0871, Japan; 2 Graduate School of Medicine, Osaka university, Osaka, 565-0871, Japan; 3 The Institute of Medical Science, The University of Tokyo, Tokyo 108-8639, Japan; 4 Center for Infectious Disease Education and Research, Osaka university, Osaka 565-0871, Japan

**Keywords:** spermiogenesis, *Ccdc188*, acephalic spermatozoa, connecting piece, male infertility

## Abstract

Acephalic spermatozoa syndrome (ASS) represents a rare genetic and reproductive disease, which is defined as semen composed of mostly headless spermatozoa. The connecting piece in the neck region, also known as the head-to-tail coupling apparatus (HTCA), plays a crucial role in the tight linkage between the sperm head and tail. Dysfunction of this structure can lead to separation of sperm heads and tails, and male infertility. Using the mouse as an experimental model, several proteins have been identified as associated with the HTCA and disruption of these proteins causes acephalic spermatozoa. However, the molecular mechanism underlying this morphologic anomaly and HTCA remains elusive. In this study, we focused on coiled-coil domain containing 188 (*Ccdc188*), which shows testis-enriched expression. To elucidate the physiological role of CCDC188, we generated a knockout (KO) mouse line using the CRISPR/Cas9 system. *Ccdc188* KO male mice were sterile, indicating that CCDC188 is indispensable for male fertility. Most *Ccdc188*-null spermatozoa were acephalic. Transmission electron microscopy revealed that while the sperm HTCA could assemble properly without CCDC188, the HTCA failed to attach to the nucleus during spermiogenesis, leading to sperm head and neck separation. In addition, we found almost all of the spermatozoa in the cauda epididymis lacked a mitochondrial sheath. Taken together, we demonstrated that CCDC188 plays a crucial role in forming a tight sperm head-neck junction.

## Summary sentence

*Ccdc188* is essential for a tight linkage between the sperm head and neck, and loss of CCDC188 leads to infertility in male mice due to acephalic spermatozoa with motility defects.

## Introduction

The structural and functional integrity of spermatozoa is essential for male fertility, and any defects in spermatozoa might be associated with male infertility [1]. Acephalic spermatozoa syndrome (ASS), characterized by headless spermatozoa in the semen, is a rare but severe type of teratozoospermia that leads to male infertility [2,3]. Following a series of studies related to this disease [2-4] and a deeper comprehension of spermatogenesis, the terms “acephalic spermatozoa” and “decapitated spermatozoa” were introduced to characterize this morphologic anomaly [3].

The sperm head-tail coupling apparatus (HTCA), also called the connecting piece or sperm neck, is a centrosome-based structure facilitating the firm attachment between the sperm head and flagellum. The HTCA develops from the centrosome and consists of proximal centrioles, distal centrioles and pericentriolar material, including basal plate, segmented columns and capitulum [5,6]. During spermiogenesis, the proximal centriole attaches to the nucleus to form the implantation fossa, which is located opposite the acrosome [7]. Subsequently, the segmented columns and the capitulum assemble to form a connecting piece in this region [8,9]. The attachment of the HTCA to the nuclear membrane is essential for establishing a tight connection between the sperm head and tail. Any disruption in this process can lead to male infertility with acephalic spermatozoa [5]. Although ASS and HTCA have been characterized for decades, the molecular mechanisms of ASS and HTCA are recent areas of study.

Coiled-coil domain-containing 188 (CCDC188) belongs to the CCDC family which consists of proteins with coiled-coil domains that contain two to six helices [10]. A previous study generated a knockout (KO) mouse line targeting testis-enriched coiled-coil domain containing 42 (*Ccdc42*) and confirmed that deletion of this gene caused sterility in male mice with malformation of the HTCA and the sperm tail [11]. In a previous study, a homozygous nonsense variant in *CCDC188* was detected in an ASS patient, and mice lacking *Ccdc188* exhibited male infertility with acephalic spermatozoa [12]. This study elucidated that CCDC188 was localized to the HTCA of human and mouse spermatozoa. In addition, this study revealed that CCDC188 interacts with SUN5 and PMFBP1, both of which disruptions are associated with acephalic spermatozoa [12]. However, this study lacks detailed observations during spermiogenesis. Here, we generated *Ccdc188* KO mice using the CRISPR/Cas9 system and observed HTCA formation using transmission electron microscopy (TEM) to further analyze how CCDC188 functions in HTCA formation during spermiogenesis.

## Materials and Methods

### Animals

All animal experiments were approved by the Animal Care and Use Committee of the Research Institute for Microbial Diseases, Osaka University. Animals were housed in a temperature-controlled environment with 12 h light cycles and free access to food and water. B6D2F1 (C57BL/6 × DBA2), ICR, or C57BL6/J mice were used as embryo donors, foster mothers, or RNA extraction, respectively. These animals were purchased from CLEA Japan, Inc. (Tokyo, Japan) or Japan SLC (Shizuoka, Japan).

### Isolation of RNA and RT-PCR

Isolation of RNA and RT-PCR was performed as previously described [13]. RNA was isolated and purified from multiple adult tissues and testes from 0 to 35 days after birth from C57BL/6N mice with TRIzol (Thermo Fisher Scientific, Waltham, MA, USA). Reverse transcription was performed using SuperScript IV Reverse Transcriptase (Thermo Fisher Scientific) with an oligo (dT) primer. PCR was carried out using KOD FX Neo (TOYOBO, Osaka, Japan). The primers used in this study are listed in Table S1.

### Generation of *Ccdc188* KO mice

**Figure 1.**
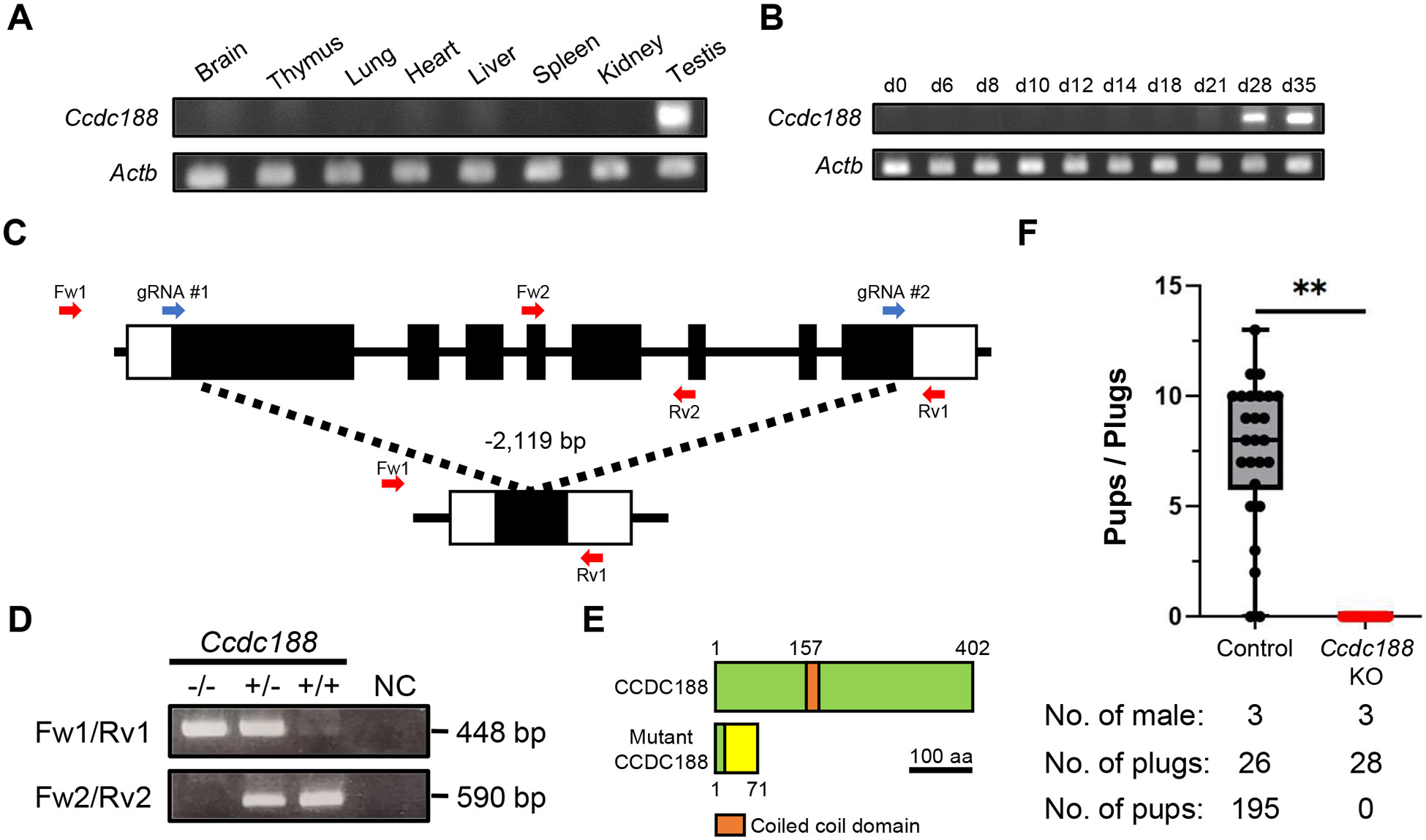
*Ccdc188* KO male mice generated by CRISPR/Cas9 are sterile (A) RT-PCR for *Ccdc188* from various mouse tissues. *Actb* was used as a loading control (*N* = 2). (B) RT-PCR for *Ccdc188* using RNAs obtained from various postnatal mouse testes. *Actb* was used as a loading control (*N* = 2). (C) KO strategy for generating *Ccdc188* KO mice. The upper and bottom panels show diagrams for WT and KO alleles, respectively. Two gRNAs (blue arrows) were designed to target exon 1 and 8. Fw1 and Fw2 are forward primers for genotyping. Rv1 and Rv2 are reverse primers for genotyping. Our study generated a *Ccdc188* KO mouse line with a 2,119-bp deletion. (D) Genotyping of *Ccdc188* KO mutant mice. Fw1/Rv1 and Fw2/Rv2 primers in Fig. 1C were used. (E) A predicted structure of mouse CCDC188 and mutant CCDC188 proteins. Mutant CCDC188 lacks the coiled-coil domain (157 to 192 aa). Yellow box indicates the amino acid sequence that does not match the proper sequence. (F) Number of pups born per plug detected. Three males each for control and *Ccdc188* KO were mated with three WT females per male (** *P* < 0.01, Student’s t-test, *N* = 3).

*Ccdc188* KO mice were generated as previously described [14]. We designed two guide RNAs (gRNAs) to recognize exon 1 and exon 8 to remove most of the coding sequence (Fig. 1C). The crRNA sequences used in this study were 5’- AAACTGGGGCCAGAGGACGC −3’ and 5’- CCCCTTCTTGCGCCTCGAAG −3’. Synthesized crRNAs (Merck, Darmstadt, Germany), tracrRNA (Merck) and CAS9 protein (Thermo Fisher Scientific) were incubated to make the CAS9 ribonucleoprotein (RNP) complex. The obtained RNP complex was electroporated into fertilized eggs using a NEPA21 electroporator (NEPA GENE, Chiba, Japan). Fertilized eggs that had been electroporated were transplanted into the oviducts of pseudopregnant females. The obtained pups were genotyped by PCR and then subjected to Sanger sequencing to verify the deleted sequence.

### Genotyping analysis

Genotyping PCR was performed using KOD FX Neo. The primers used in this study are listed in Table S1.

### In vivo fertility test

To confirm the fertility of *Ccdc188* KO male mice, natural mating tests were conducted. Three male mice were individually caged with three B6D2F1 females for 2 months. Both plug and pup numbers were checked at approximately 10 AM every weekday to determine the number of copulations and litter size. To confirm the fertility of *Ccdc188* KO female mice, sexually mature wild-type (WT) or KO female mice were caged individually with a B6D2F1 male for at least 8 weeks. Male mice were removed after the mating period, and females were kept for another three weeks to count the final litters. The number of pups and litters were counted every weekday morning.

### Morphological and histological analysis

Spermatozoa were collected from the cauda epididymis, and suspended in TYH medium [15]. A sperm suspension was mounted on MAS-coated glass slides (Matsunami Glass, Osaka, Japan), and a cover slip (Matsunami) was added. Sperm morphology was observed using a BX53 microscope (Olympus, Tokyo, Japan).

Morphological and histological analysis of testis was conducted as previously described [16]. Male mice were euthanized and testes were dissected. After measuring the testicular weight, testes and epididymides were fixed with Bouin’s fixative (Polysciences, Warrington, PA, USA). Fixed testes and epididymides were embedded in paraffin, sectioned, rehydrated, and treated with 1% periodic acid for 10 min, followed by treatment with Schiff's reagent (Wako, Osaka, Japan) for 20 min. The sections were stained with Mayer’s hematoxylin solution prior to imaging and observed using a BX53 microscope (Olympus).

### Sperm head/flagellum counting

Testes, caput, and caudal epididymides were carefully dissected in PBS and the contents were released into TYH medium [15]. A small aliquot (~5 μL) was spread onto a MAS-coated glass slide (Matsunami), and a cover slip (Matsunami) was added, followed by examination under a BX53 microscope (Olympus). The total number of intact spermatozoa, separated sperm heads, and separated flagella was counted.

### Sperm motility analysis

Sperm motility analysis was conducted as described previously [17]. Cauda epididymal spermatozoa were suspended and incubated in TYH medium that can induce sperm capacitation [15]. Sperm motility was then measured using the CEROS II sperm analysis system (software version 1.5; Hamilton Thorne Biosciences, Beverly, MA, USA). The motility of epididymal spermatozoa was recorded after 10 min and 2 h of incubation in TYH medium.

### Immunofluorescence

Immunofluorescence analysis of testes was performed using cryosections as previously described [18]. Testes were fixed with 4% paraformaldehyde, embedded in OCT compound (Sakura Finetek, Tokyo, Japan), and 10 μm sections were prepared with a cryostat (CryoStar NX70, Thermo Fisher Scientific). The sections were subjected to antigen retrieval, permeabilization, and blocking. Then, the sections were incubated overnight at 4 °C with primary antibodies. After three washes, the appropriate Alexa Fluor-conjugated secondary antibodies (Thermo Fisher Scientific) and Alexa Fluor-conjugated lectin PNA (Thermo Fisher Scientific) were added to the slides and incubated. The sections were stained with Hoechst 33342 (Thermo Fisher Scientific) for visualizing nuclei and coverslipped with Immu-Mount (Thermo Fisher Scientific).

Immunofluorescence analysis of spermatozoa was performed as previously described [19]. Spermatozoa were suspended in PBS, smeared on microscope slides, dried at 37 °C for 15 min, fixed with 4% PFA, blocked with 3% BSA for 1 h and immunostained with primary antibodies. Goat-anti-rabbit or -mouse Alexa Fluor-conjugated secondary antibodies (Thermo Fisher Scientific) were used as the secondary antibody. The samples were then stained with Hoechst 33342 (Thermo Fisher Scientific) for visualizing nuclei, and coverslipped with immu-Mount (Thermo Fisher Scientific).

Microscopic images were obtained using a Nikon Eclipse Ti microscope connected to a C2 confocal module (Nikon, Tokyo, Japan) or a BX53 microscope (Olympus). Fluorescent images were false-colored and cropped using ImageJ software (version 2.0.0, NIH, Bethesda, MD, USA). The antibodies used in this study are listed in Table S2.

### Ultrastructural analysis using transmission electron microscopy

Ultrastructural analysis using TEM was conducted as previously described [16].

### Statistical analyses

Statistical analyses were performed using a two-tailed student's t-test (** *P* < 0.01) by GraphPad Prism 9.5.0 (GraphPad, San Diego, CA, USA). Data were plotted graphically using Box-and-Whisker plot.

## Results

### *Ccdc188* KO male mice are sterile

To investigate the expression profile of *Ccdc188*, we performed reverse transcription polymerase chain reaction (RT-PCR) using RNAs extracted from multiple mouse tissues. RT-PCR demonstrated abundant expression of *Ccdc188* in the testis, not in other tissues (Fig. 1A), which is consistent with the Mammalian Reproductive Genetics Database [20]. To determine at which stage *Ccdc188* starts to express, RT-PCR was conducted using RNAs obtained from postnatal testicular tissues. *Ccdc188* starts to express from day 28 (Fig. 1B), corresponding to the onset of elongating spermatids [21]. On the other hand, a published genetic database indicated that *Ccdc188* expressed in round spermatids predominantly [20]. These data suggest that *Ccdc188* is a testis-specific gene which may play a role in spermiogenesis.

To elucidate the in vivo functions of *Ccdc188*, we employed the CRISPR/Cas9 system to generate *Ccdc188* KO mice. The mouse *Ccdc188* gene contains eight exons, and the encoded protein contains 402 amino acids. We designed the gRNAs near the start codon and stop codon to remove almost all of the whole coding region (Fig. 1C). Of the 56 fertilized eggs that had been electroporated with CRISPR/Cas9 targeting *Ccdc188*, 51 embryos were transferred to pseudopregnant mothers. A total of 4 potential founder mice (F0) were born, and 2 pups possessed the desired mutation. A mutant line with a 2,119 bp deletion in the *Ccdc188* gene was used for this study (Fig. 1C, D). CCDC188 was predicted to contain a coiled-coil domain [amino acids (aa) 157 to 192], and it was removed in *Ccdc188* KO mouse line (Fig. 1E). *Ccdc188* KO mice were viable and did not show overt abnormalities. Whereas *Ccdc188* KO adult females were fertile (Fig. S1A), *Ccdc188* KO male mice showed sterility. Even though 28 copulatory plugs were observed, no pups were born from *Ccdc188* KO male mice parings (Fig. 1F), indicating the indispensable role of *Ccdc188* in male fertility.

### Loss of CCDC188 results in acephalic spermatozoa without a mitochondrial sheath during spermiogenesis

**Figure 2.**
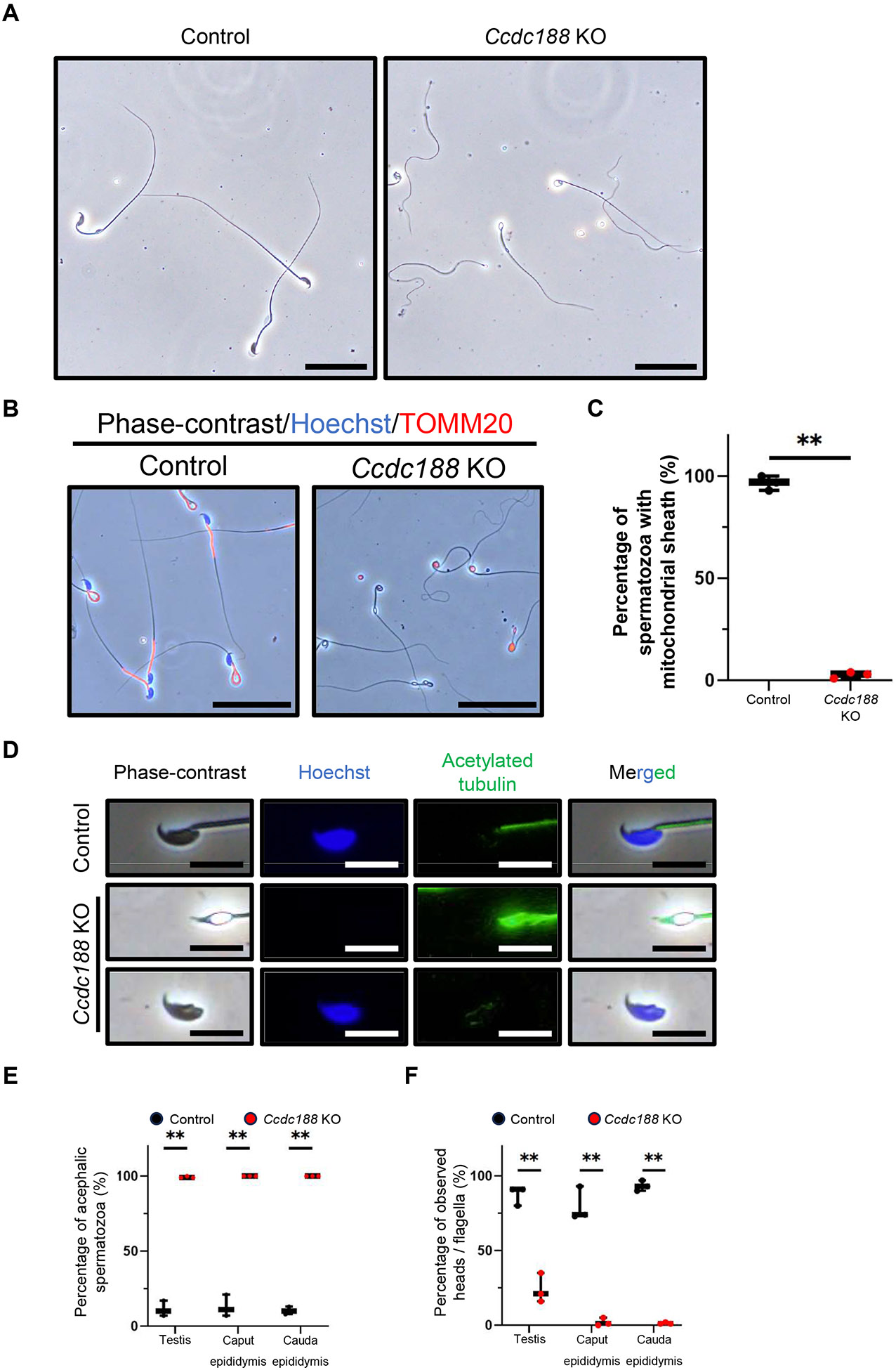
Loss of CCDC188 results in acephalic spermatozoa without a mitochondrial sheath during spermiogenesis (A) Observation of spermatozoa obtained from cauda epididymis. Almost all spermatozoa are abnormal. Scale bars: 50 μm. (B) Immunofluorescent analysis of control and *Ccdc188* KO spermatozoa by TOMM20 (red) and Hoechst 33342 (blue) to visualize mitochondria and nuclei, respectively. The mitochondrial sheath was hardly found in *Ccdc188* KO mice. Scale bars: 50 μm. (C) Graph indicates frequencies of spermatozoa with a mitochondrial sheath collected from the cauda epididymis (** *P* < 0.01, Student’s t-test, *N* = 3). (D) Immunofluorescent analysis of spermatozoa obtained from control and *Ccdc188* KO cauda epididymis. Spermatozoa were stained with acetylated tubulin (green) to visualize flagella. Hoechst 33342 (blue) was used to visualize the nuclei. Hoechst signal can not be found in most *Ccdc188*-null spermatozoa. Additionally, just a few spermatozoa exhibited separated sperm heads without a flagellum. Scale bars: 10 μm. (E) Graph indicates frequencies of acephalic spermatozoa collected from the testis, caput epididymis, and cauda epididymis (** *P* < 0.01, Student’s t-test, *N* = 3). (F) The graph indicates the percentage of observed sperm heads per flagella collected from the testis, caput epididymis, and cauda epididymis (** *P* < 0.01, Student’s t-test, *N* = 3).

To reveal the cause(s) of male infertility in *Ccdc188* KO mice, we observed spermatozoa obtained from *Ccdc188* KO cauda epididymis. Most of the spermatozoa we observed showed abnormalities in the head region (Fig. 2A). When we measured the length of sperm tails, sperm tails from *Ccdc188* KO mice were significantly shorter than those from control males, and the mean tail length of control spermatozoa and *Ccdc188*-null spermatozoa was 116.0 μm and 92.8 μm, respectively (Fig. S1B). Because the average length of midpiece and the rest of tail (principal piece and endpiece) are around 23 μm and 95 μm in mice [22], these results foresee the lack of midpiece in *Ccdc188* KO spermatozoa. We then immunostained the spermatozoa with the mitochondrial marker, TOMM20. As expected, almost no *Ccdc188* KO spermatozoa (2.7%) had a mitochondrial sheath (Fig. 2B and C). Then, immunofluorescence using anti-acetylation of α-tubulin (αK40, acetylated tubulin) and Hoechst 33342 staining was conducted for further evaluation of sperm morphology. Most of the *Ccdc188*-null spermatozoa were unable to be stained by Hoechst, which indicates that most KO spermatozoa are headless or acephalic spermatozoa (Fig. 2D). Additionally, just a few sperm heads with normal morphology were observed, but most of them does not have flagellum (Fig. 2D). We then collected spermatozoa from testes, caput epididymides, and cauda epididymides to reveal when acephalic spermatozoa emerge. From our results, the percentage of acephalic spermatozoa in control testis, caput epididymis, and cauda epididymis was 11.3%, 13.0% and 10.3%. However, in *Ccdc188* KO mice, the number of acephalic spermatozoa increased to 99.3%, 100%, and 100% in testis, caput epididymis, and cauda epididymis (Fig. 2E and S1C). These results indicate that separation of the sperm head from the flagella occurs in the testis. Moreover, more sperm flagella than sperm heads were present in both KO testes and epididymides compared with control samples (Fig. 2F). In addition, sperm heads were rarely found in epididymides, but a few sperm heads can be isolated from the testes (Fig. 2F and S1C). These results imply that spermatid heads are removed by Sertoli cells and only sperm flagella transit into the epididymis in many cases. To check the motility of the acephalic spermatozoa, we assessed sperm motility by computer-assisted spermatozoa analysis (CASA). CASA revealed a significant reduction in the motility of *Ccdc188* KO acephalic spermatozoa (Fig. S1D and E). In summary, disruption of *Ccdc188* leads to the production of acephalic spermatozoa without a mitochondrial sheath and abnormal sperm motility, which are the causes of *Ccdc188* KO male infertility.

**Figure 3.**
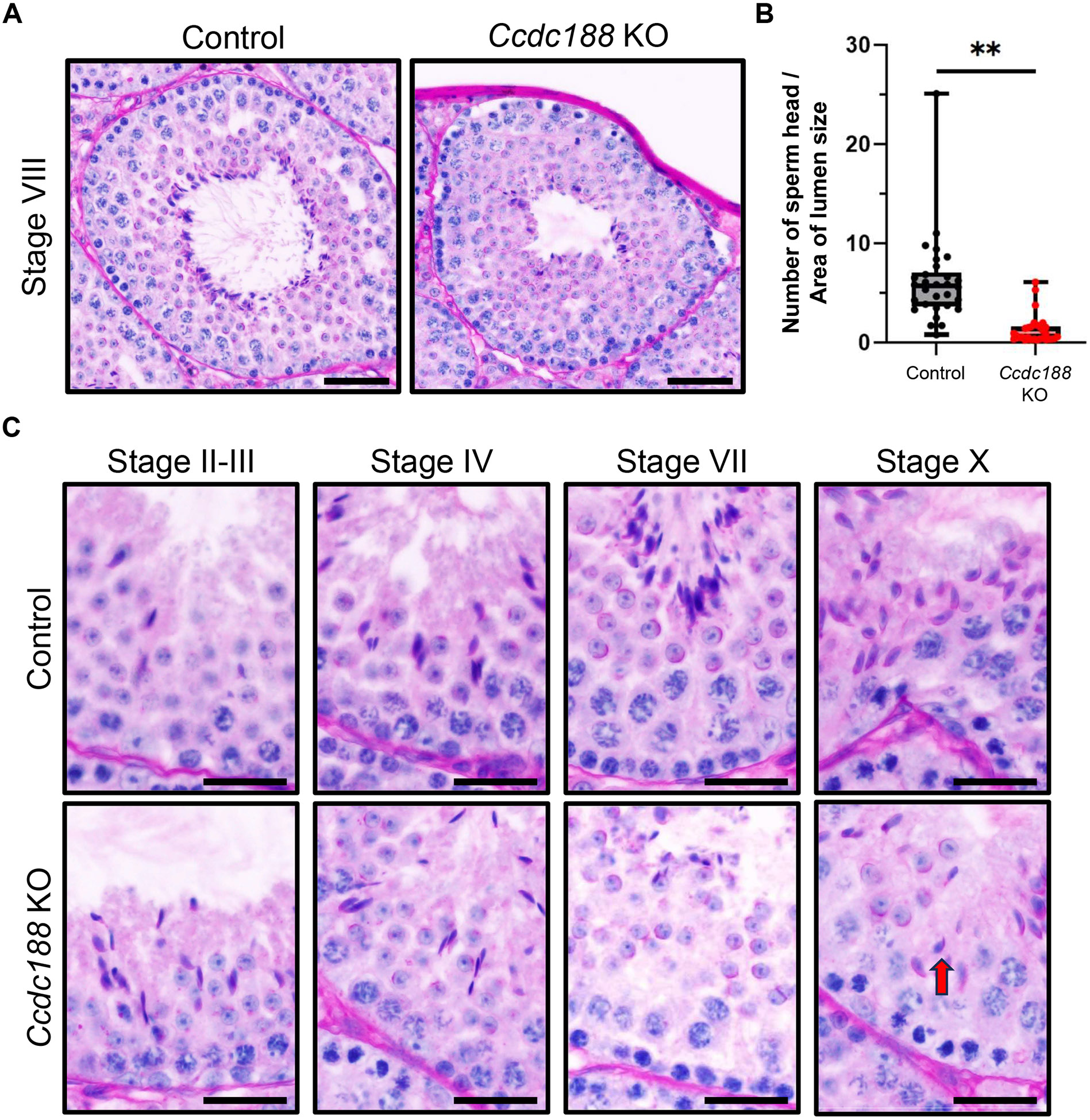
Cross sections of seminiferous tubules from *Ccdc188* KO testis (A, B, C) Hematoxylin and PAS staining of testicular sections of adult control and *Ccdc188* KO mice. (A) There are fewer elongated sperm heads in *Ccdc188* KO testis. Scale bars: 50 μm. (B) Graph indicates the number of sperm heads aligned along the lumen (** *P* < 0.01, Student’s t-test, *N* = 3). (C) Mature sperm heads (red arrow) can be observed in the seminiferous tubules after stage VIII in *Ccdc188* KO testis. Scale bars: 20 μm.

Mature spermatids are released into the seminiferous tubule lumen by spermiation [23]. The released spermatozoa traverse the efferent ducts and finally reach the cauda epididymis, where they are stored before ejaculation [24]. When we observe the spermatozoa inside the caput and cauda epididymis by hematoxylin and periodic acid-Schiff (PAS) staining, spermatozoa within the control epididymis exhibited more intense hematoxylin staining than those in the KO epididymis, suggesting that sperm heads in the *Ccdc188* KO epididymis are much less abundant (Fig. S2A) which is consistent with the previous results (Fig. 2E and F). Then, we examined the *Ccdc188* KO testis at both gross and histological levels. No overt differences in testis size and appearance were observed between control and KO mice (Fig. S2B and C). Next, we observed histology of testis cross-sections with hematoxylin and PAS staining. Step 16 spermatids are the most developed spermatids in the testis which are aligned along the lumen to be released and transferred to the epididymis. However, the numbers of sperm heads in the KO testes were low compared with control (Fig. 3A and B). Then we carefully examined the cross-sections in testes and found that the mature sperm heads could still be observed at stage X seminiferous tubules in the *Ccdc188* KO testes (Fig. 3C). These results indicate a reduction in the number of released sperm heads observed in both the testes and epididymides of *Ccdc188* KO mice, which are consistent with the decreased count of sperm heads in both the testicular and epididymal sperm suspensions from *Ccdc188* KO mice (Fig. 2E and F). We also quantified the lumen size of the seminiferous tubules, but it was comparable between control and *Ccdc188* KO testis (Fig. S2D). To reveal the subcellular localization of CCDC188, we generated two kinds of anti-CCDC188 antibodies targeting 81-92 aa and 111-124 aa, but both failed to work for immunoblotting and immunofluorescence analysis.

### The disruption of *Ccdc188* impaired head-to-neck anchoring in spermatids

A structure known as the sperm HTCA plays a crucial role in integrating the sperm head and tail together [5]. Defects in HTCA formation lead to the separation of the sperm head from the tail, resulting in ASS that leads to male infertility [3,25]. To gain insights into HTCA dynamics in *Ccdc188* KO mice, we conducted a close examination of this structure using TEM.

**Figure 4.**
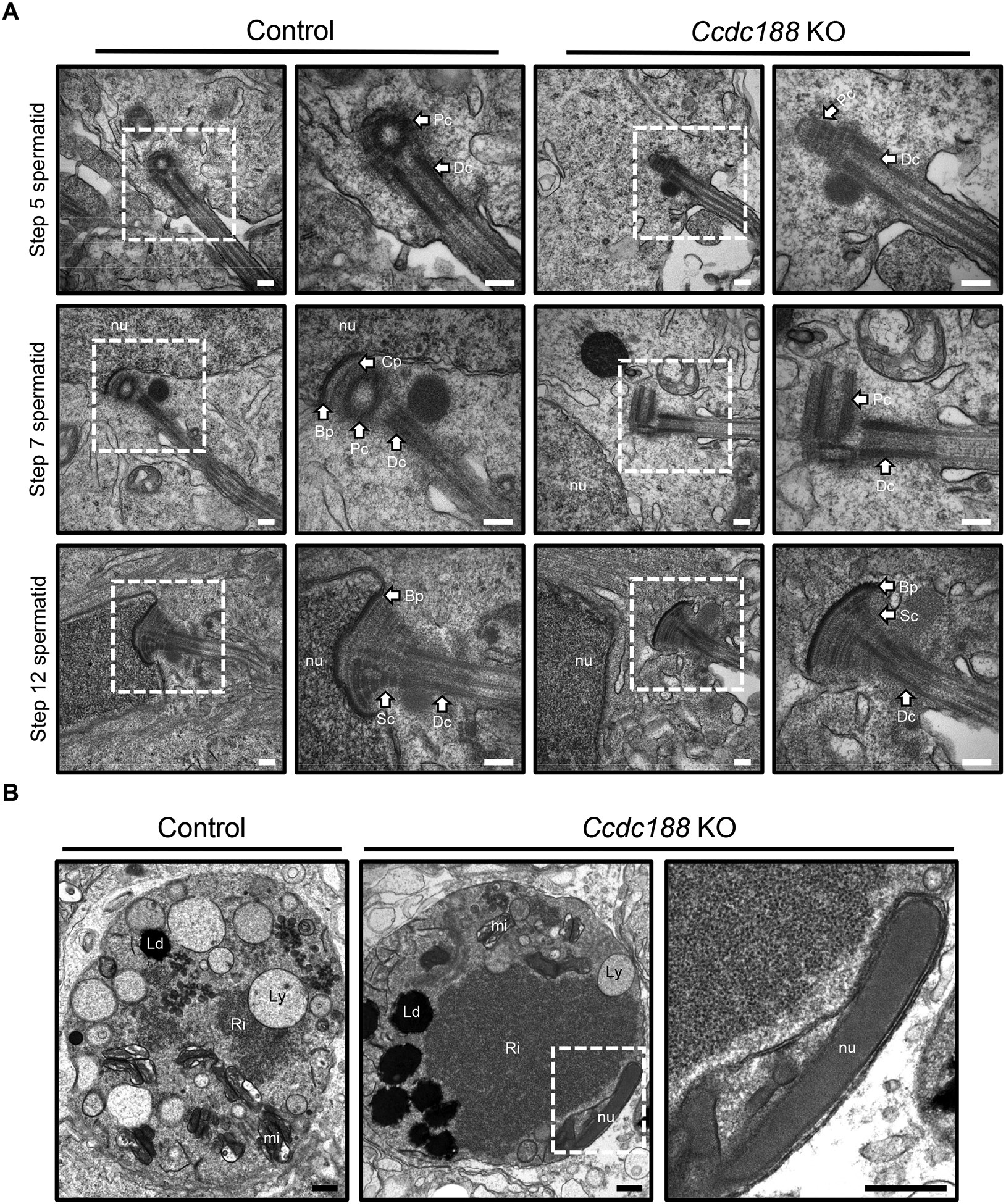
Disruption of CCDC188 impairs head-to-neck anchoring in spermatids (A) TEM analyses of the stepwise development of the head-to-tail coupling apparatus (HTCA) in control and *Ccdc188*-null spermatozoa. In the step 5 spermatid, the centrioles do not attach to the nucleus in both control and *Ccdc188*-null spermatids. In the following developmental stages, though the coupling apparatus can be assembled, it cannot be tightly attached to the nuclear envelope in *Ccdc188*-null spermatids (*N* = 2). Scale bar: 200 nm. Bp: Basal plate, nu: nucleus, Sc: Segmented column, Cp: Capitulum, Pc: Proximal centriole, Dc: Distal centriole. (B) Ultrastructural images of the residual body in control and *Ccdc188* KO testis. Nuclei were incorporated into residual bodies in *Ccdc188* KO mice (*N* = 2). Scale bar: 500 nm. nu: nucleus, Ld: Lipid droplets, Ly: lysosomes, mi: mitochondrial, Ri: Ribosome complex.

Axonemal microtubules begin to elongate from the distal centriole at the cell surface during steps 2-3, but the centriole pairs do not attach to the nuclear membrane at these steps [26-28]. The attachment of centriole pairs to the nuclear envelope can be observed in step 6–7 spermatids [28,29]. As expected, the centriole pairs with axonemal microtubules did not localize to the caudal nuclear pole in both control and *Ccdc188* KO spermatids in step 5 (Fig. 4A, top lane). In step 7 spermatids of control mice, the proximal centriole attached to the caudal side of the nucleus, whereas in *Ccdc188* KO mice, the centriole pair was apart from the nucleus (Fig. 4A, middle lane). In step 12 spermatids, while the centriole pair in control testis was tightly attached to the nuclear envelope, that of *Ccdc188* KO was far away from the nuclear envelope although the HTCA was fully formed (Fig. 4A, bottom lane). Also, we observed that sperm heads were abnormally present within residual bodies in *Ccdc188* KO testes (Fig. 4B). These findings suggest that the separated sperm heads are likely engulfed and absorbed by Sertoli cells, which leads to the decreased number of sperm heads in the KO testis and epididymis.

Since shorter tails and absence of midpiece were observed in *Ccdc188* KO mice (Fig. S1B and Fig. 2C), we sought to investigate whether the disrupted head-to-neck connection could cause abnormal flagella development. To confirm this hypothesis, we performed an immunofluorescent analysis of the testis using an anti-acetylated tubulin antibody. Spermatid flagella were observed in *Ccdc188* KO testis, but most were located near round spermatids and few were found in the lumen of seminiferous tubules (Fig. S3A). We then performed TEM analysis to analyze the sperm flagella in more detail. We could observe normal “9+2” axonemal microtubule structures in the flagellum in KO spermatids with normal mitochondrial and fibrous sheaths (Fig. S3B). However, an axoneme wrapped by a mitochondrial sheath could also be observed inside the residual body in *Ccdc188* KO mice (Fig. S4A), which is thought to be the cause of mitochondrial sheath absence (Fig. 2B and C). The annulus, the border between the midpiece and the principal piece in spermatozoa, migrates toward the border between midpiece and principal piece from the neck region in late spermiogenesis, and the mitochondria are assembled as a helical structure proximal to the annulus [30]. In both control and *Ccdc188* KO mice, the annulus could be observed near the HTCA of step 12 spermatids and at the distal end of the midpiece in step 15 spermatids (Fig. S4B), which indicates that *Ccdc188* KO spermatids undergo annulus migration normally. However, when we observed step 16 spermatids, the annulus was visible between the midpiece and principal piece in control, but the annulus with no principal piece was observed in *Ccdc188*-null spermatids (Fig. S4B). These results indicate that the *Ccdc188*-null flagella broke between the midpiece and principal piece after mitochondrial assembly and only the principal piece got released to the epididymis. We then employed the SEPTIN4 antibody, a marker of the annulus. SEPTIN4 signals were detected at the border between the midpiece and principal piece of the control flagella. In contrast, SEPTIN4 signals were not observed or observed at the edge of the flagella in the *Ccdc188*-null spermatozoa (Fig. S5), which indicates that the principal piece with/without annulus is released into the lumen and migrates to the epididymis. These results suggest that even though *Ccdc188* KO spermatids can form normal axonemal structures and assemble mitochondria in the midpiece, disruption of *Ccdc188* leads to not only the absence of the sperm head but also the absence of the midpiece.

## Discussion

ASS, characterized by semen predominantly containing spermatozoa without heads and present in some infertile men, has been reported for decades [2,3,31]. The HTCA, located in the neck region of the mammalian spermatozoa, is essential for integrating the sperm head and tail [8,32]. HTCA comprises a structure lining the implantation fossa known as the basal plate, along with a dense, convex articular region called the capitulum. Extending posteriorly from the capitulum are nine cylindrically segmented columns [25]. At the caudal end, one of the nine outer dense fibers is continuous with each segmented column, which is associated with peripheral microtubular doublets of the growing axoneme [5]. Additionally, the distal centriole is located at the base of the axoneme oriented approximately perpendicular to the proximal centriole [33,34]. Despite a detailed description of these structures over decades, their molecular composition and assembly properties remain unknown. Wang *et al.*, revealed that one of the infertile patients with ASS has a homozygous nonsense variant in *CCDC188,* and also *Ccdc188* KO male mice are infertile with acephalic spermatozoa [12]. In addition, they described that CCDC188 was localized to the HTCA of human and mouse spermatozoa [12]. However, how CCDC188 is involved in HTCA development during spermiogenesis is still unknown.

Our analysis of *Ccdc188* KO mice generated independently of the previous report [12] indicates that their phenotypes are similar. Both previous reports and our study indicate that loss of functional CCDC188 can cause acephalic spermatozoa in mice. However, there are some differences between our study and the previous one. In the previous study, ASS syndrome of infertile patients with nonsense variant in *CCDC188* was caused by a break between the nucleus and the proximal centriole [12]. In our study, we revealed that *Ccdc188* KO spermatids failed to link the head and HTCA during spermiogenesis without detectable problems with HTCA formation by TEM analysis of spermatids (Fig. 4A). Moreover, we demonstrated that *Ccdc188*-disrupted spermatozoa are shorter than control spermatozoa due to the loss of the midpiece (Fig. 2B, 2C, S4B, and S5), which is a new finding compared with the previous study. The break between the midpiece and principal piece (Fig. S4B) may be caused by the force resulting from hydrostatic pressure within the seminiferous tubule [35]. Then, sperm heads and midpieces are phagocytosed by Sertoli cells as residual bodies (Fig. 4B and S4A), and other flagella move to the epididymis (Fig. 2A). In addition, we discovered that the failure of the attachment of sperm head and HTCA occurs in *Ccdc188* KO mice during spermiogenesis (Fig. 4A).

In recent decades, several studies have revealed that ASS with familial clustering, indicating a specific genetic basis for this syndrome [7,36-39]. Many mouse models have been identified to exhibit the acephalic spermatozoa phenotype [40-46], among which Sad1 and UNC84 domain containing 5 (SUN5) localizes on the nuclear envelope of the spermatids and later accumulates in the neck region of the spermatozoa [42]. Disruption of SUN5 in mice demonstrated that HTCA can be successfully assembled without SUN5, but cannot attach to the nuclear envelope [42,47]. Similar to *Sun5* KO mice, HTCA in *Ccdc188* KO mice was fully developed with intact segmented columns, capitulum, and basal plate, but the basal plate was not attached to the nuclear envelope (Fig. 4A). In *Sun5* KO mice, the acephalic sperm heads can be observed surrounded by Sertoli cells near the basal membrane [47], which corresponds with our *Ccdc188* KO results (Fig. 4B).

As mentioned above, both CCDC188 and SUN5 are essential for the head and HTCA linkage. However, there are some differences between *Ccdc188* KO and *Sun5* KO phenotypes. Nearly all of the *Ccdc188*-null spermatozoa are immotile (Fig. S1D and E), while 60% of *Sun5*-null spermatozoa can still move. *Sun5* KO sperm midpiece (or mitochondrial sheath) is observed in mature spermatozoa, but the mitochondrial sheath in *Ccdc188*-null mice is observed in the residual body in the testis (Fig. S4A), and rarely observed in mature spermatozoa (Fig. 2B and C). Therefore, loss of the midpiece may be the reason for low motility in *Ccdc188* KO mice. It has been proposed that ASS be categorized along three distinct states. The decapitation state includes (1) separation between the nucleus and centriolar region, (2) break in the midpiece, and (3) separation between the midpiece and principal piece [39]. During the spermiogenesis of *Ccdc188* KO mice, we observed the separation between HTCA and nucleus in round spermatids (Fig. 4A). After mitochondrial assembly in late elongating spermatids, the break between midpiece and principal piece is also observed by TEM analysis (Fig. S4B). Therefore, *Ccdc188* KO acephalic spermatozoa are classified in both (1) and (3) states. In contrast, *Sun5* KO spermatozoa should be classified as (1), a state separated between the nucleus and the centriolar region. These differences between CCDC188 and SUN5 can be the reasons for the different phenotypes of *Ccdc188* KO and *Sun5* KO mice but the mechanism of how CCDC188 and SUN5 work at the head-neck junction needs further studies.

While we have not determined the subcellular localization of CCDC188, the previous study has shown that CCDC188 is located specifically at the HTCA of human and mouse spermatozoa [12]. Spermatogenesis associated 6 (SPATA6) is considered to be a protein involved in the formation of the HTCA, and the absence of *Spata6* disrupts HTCA assembly [46]. Polyamine modulated factor 1 binding protein 1 (PMFBP1) is considered to localize in HTCA and involved in connecting sperm head to tail by cooperating with SUN5 and SPATA6 [48]. Because the previous study confirmed that overexpressed CCDC188 interacts with overexpressed both SUN5 and PMFBP1 in cultured cells [12], these proteins may function in HTCA attachment to the nuclear envelope cooperatively.

CCDC42 is another CCDC family protein with testis-enriched expression, and disruption of CCDC42 induces HTCA defects. However, *Ccdc42* KO mice showed multiple morphological abnormalities, including abnormal sperm heads, failure of axoneme assembly [11]. Therefore, the molecular mechanism between CCDC188 and CCDC42 is different, and future studies will help to address the functions of CCDC domain in spermiogenesis.

In this study, we validated the crucial role of CCDC188 in the linkage between the sperm head and HTCA during spermiogenesis. However, further research on the molecular mechanism by which *Ccdc188* is involved in the connection of sperm head and HTCA is needed to understand the pathogenesis. We hope that this study will contribute to the development of gene therapy for ASS associated with mutations in *Ccdc188* as well as clinical treatment of ASS.

## Supplementary Material

Supplementary figures

Supplementary tables

## Data Availability

Frozen spermatozoa from *Ccdc188* KO males (B6D2-*Ccdc188*^*em1Osb*^) were deposited at both the Riken BioResource Center, Ibaraki, Japan (RBRC number: 12258) and the Center for Animal Resources and Development (CARD), Kumamoto University, Kumamoto, Japan (CARD ID: 3489). *Ccdc188* KO mice are available through these centers.
