# Supplementary material for: Loss of CCDC188 causes male infertility with defects in the sperm head-neck connection in mice
Source: Biol Reprod. Author manuscript; Available in PMC 2025 Jan 17. (PMC11736427; doi:10.1093/biolre/ioae137)
Supplement: Supplementary figures [file NIHMS2032238-supplement-Supplementary_figures.pdf]

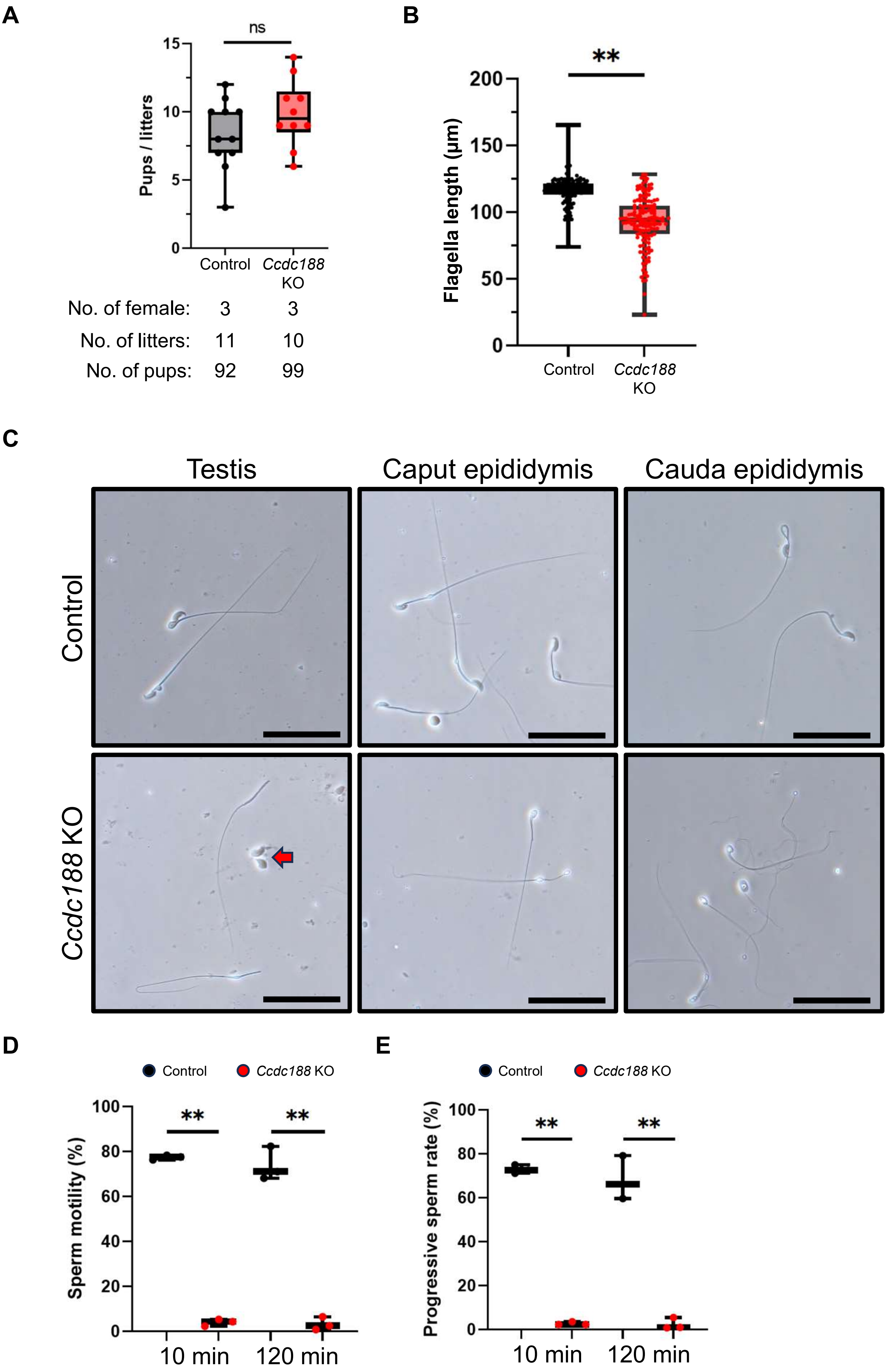
A
ns
Pups / litters
15
10
5
0
Control
Ccdc188 KO
No. of female: 3 3
No. of litters: 11 10
No. of pups: 92 99
B
**
Flagella length (μm)
200
150
100
50
0
Control
Ccdc188 KO
C
Testis
Caput epididymis
Cauda epididymis
Control
Ccdc188 KO
D
Control
Ccdc188 KO
Sperm motility (%)
100
80
60
40
20
0
**
**
10 min
120 min
E
Control
Ccdc188 KO
Progressive sperm rate (%)
100
80
60
40
20
0
**
**
10 min
120 min

Figure S1. *Ccdc188*-null spermatozoa show abnormal sperm morphology and low motility

(A) Number of pups born per litter detected. Three females each for control and *Ccdc188* KO were mated with one WT male (P = 0.1804, Student's t-test, N = 3). (B) The length of the sperm flagellum collected from the cauda epididymis. *Ccdc188*-null spermatozoa collected from the cauda epididymis were shorter than those from the control (** P < 0.01, Student's t-test, N = 3). (C) Morphology of sperm obtained from testis, caput epididymis, and cauda epididymis. Almost all *Ccdc188* KO spermatozoa are headless. Some sperm heads can be observed in the testis (red arrow) while few are seen in the epididymis in *Ccdc188* KO mice. Scale bars: 50 μm. (D, E) Motile sperm (D) and progressive sperm (E) rate from control and *Ccdc188* KO mice (** P < 0.01, Student's t-test, N = 3).

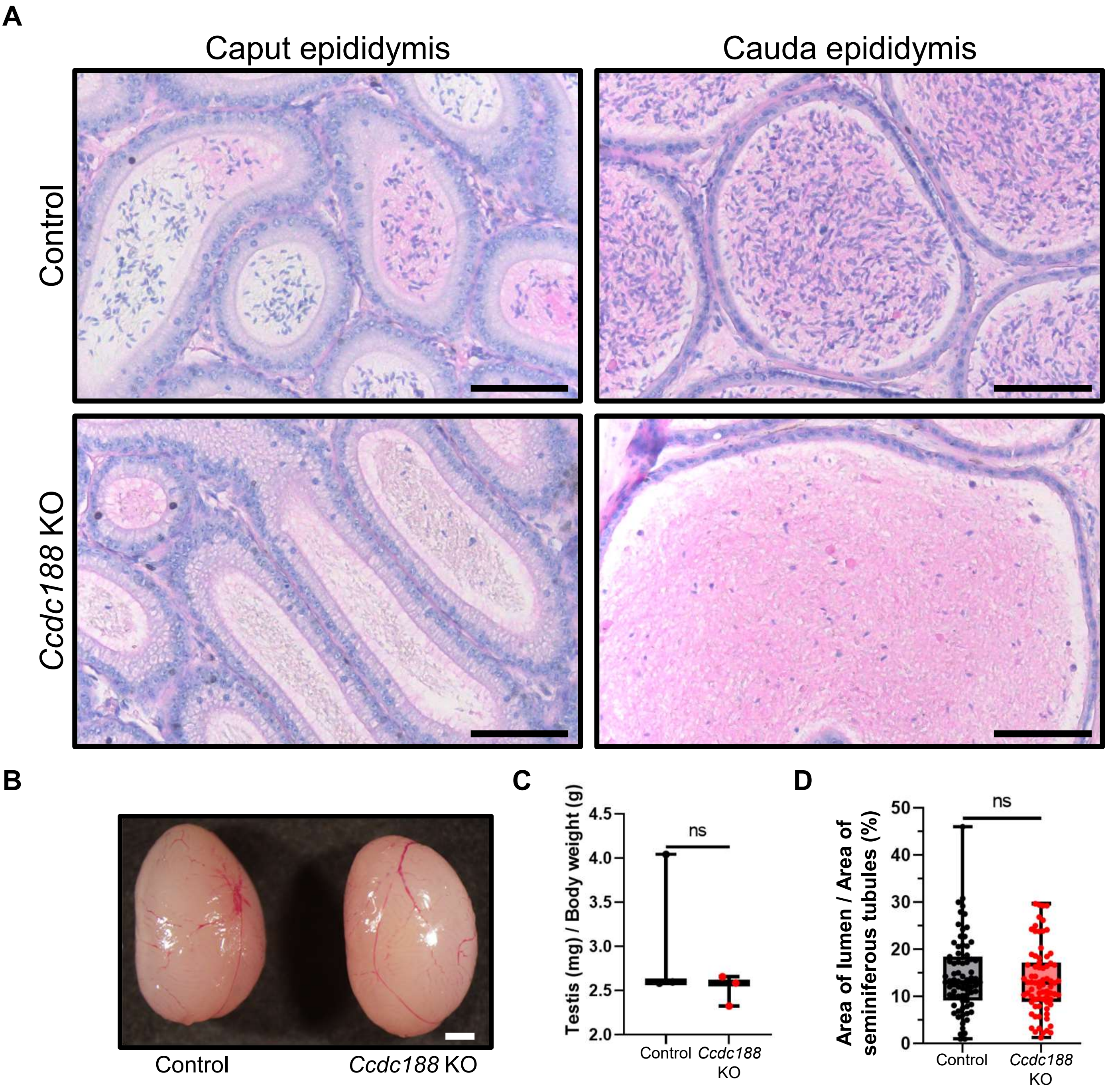

Figure S2. *Ccdc188*-null male mice have fewer sperm heads in the epididymis

(A) Hematoxylin and PAS stained sections of the caput and cauda epididymides. Sperm heads are difficult to observe in the *Ccdc188* KO epididymis. Scale bars: 100 µm. (B) Gross morphology of control and *Ccdc188* KO testes. Scale bars: 1.0 mm. (C) The average testis weight per body weight of control and *Ccdc188* KO mice (P = 0.3270, Student's t-test, N = 3). (D) Graph indicates lumen size of seminiferous tubules between control and *Ccdc188* KO testis (P = 0.5091, Student's t-test, N = 3).

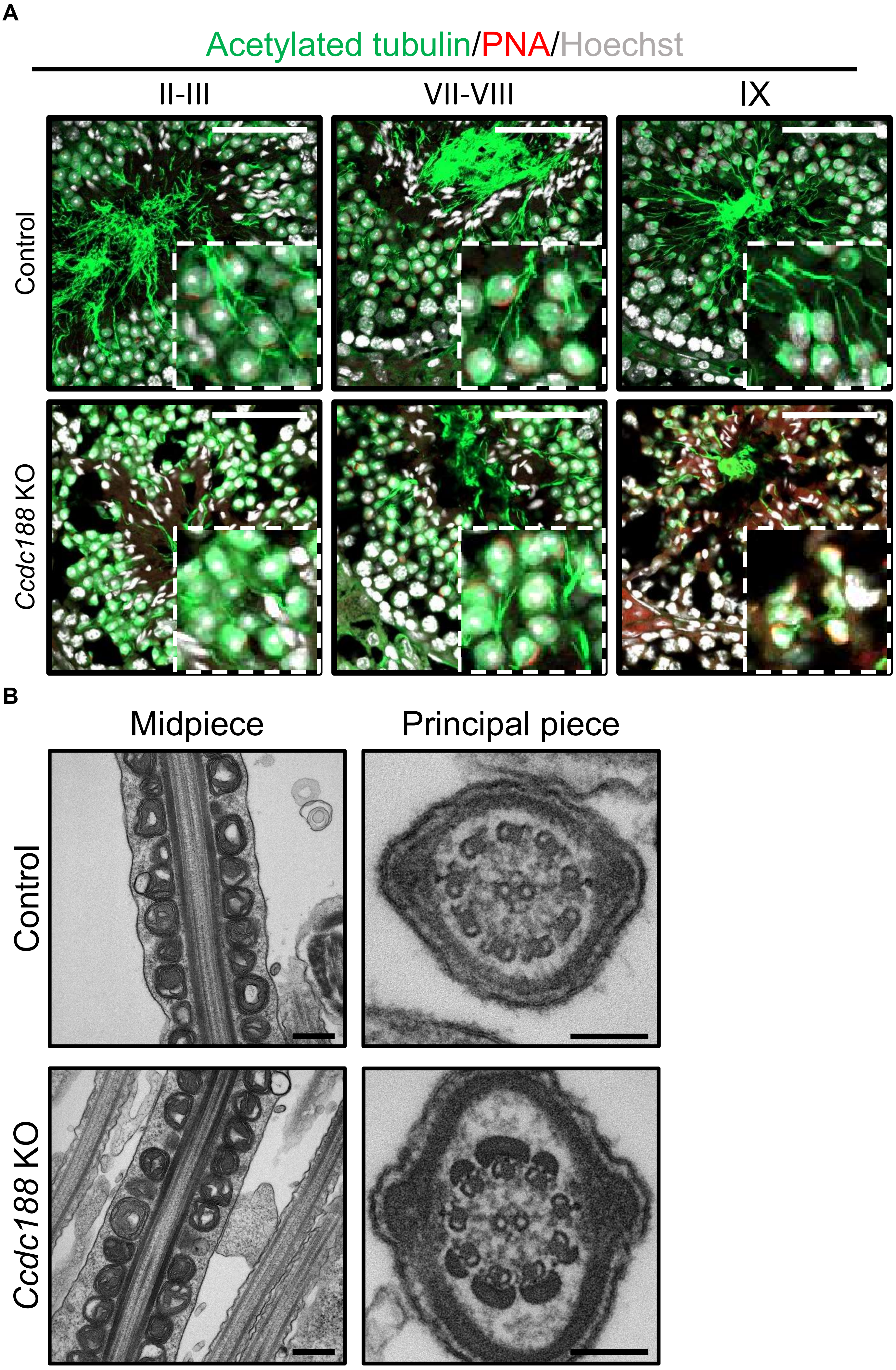
A
Acetylated tubulin/PNA/Hoechst
II-III
VII-VIII
IX
Control
Ccdc188 KO
B
Midpiece
Principal piece
Control
Ccdc188 KO

Figure S3. *Ccdc188*-null male mice have fewer sperm flagellum with normal axonemal structures in the testis

(A) Immunofluorescent analysis of flagellum in control and *Ccdc188* KO testis. Testis were stained with acetylated tubulin (green) to visualize microtubules. Hoechst 33342 (white) and PNA-lectin (red) were used to visualize the nuclei and acrosome, respectively. The number of sperm tails in the lumen of *Ccdc188* KO seminiferous tubules is dramatically lower than control. Scale bars: 20 µm. (B) Ultrastructural images of midpiece and principal piece of spermatozoa. The mitochondrial sheath was correctly localized around the axoneme and normal axonemal structures were observed in *Ccdc188* KO spermatids ($N = 2$). Scale bars: 500 nm (left) and 100 nm (right).

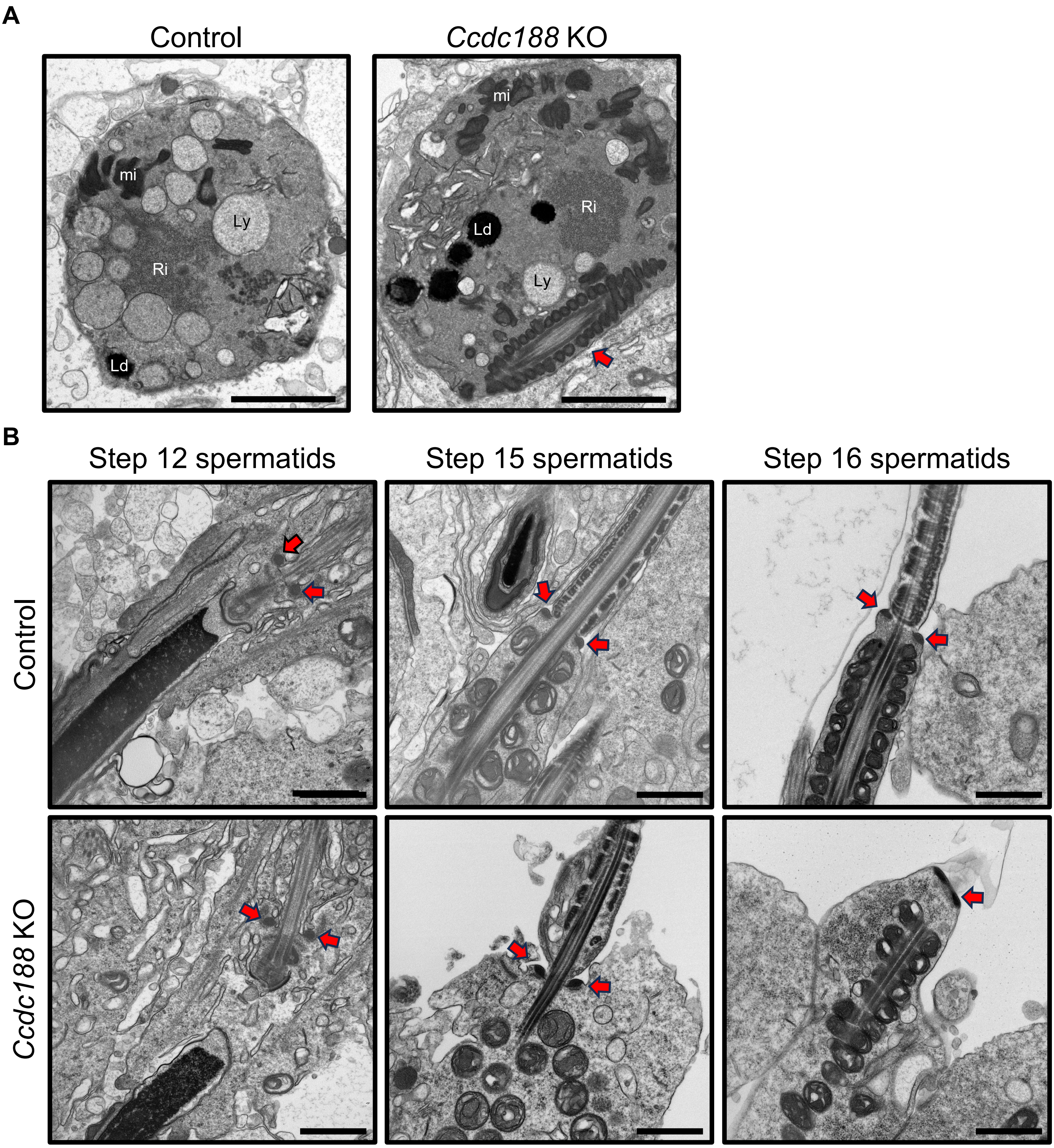

Figure S4. Separation between midpiece and principal piece can be observed in *Ccdc188* KO mice

(A) Ultrastructural images of the residual body in control and *Ccdc188* KO testis. An axoneme wrapped by a mitochondrial sheath (red arrow) was incorporated into a residual body in *Ccdc188* KO mice ($N = 2$). Scale bars: 2.0 µm. Ld: Lipid droplets, Ly: lysosomes, mi: mitochondria, Ri: Ribosome complex. (B) Ultrastructural images of annulus migration. The annulus (red arrows) migration was observed in both control and *Ccdc188* KO spermatids. However, in step 16 *Ccdc188*-null spermatids, a break between midpiece and principal piece can be observed ($N = 2$). Scale bars: 1.0 µm.

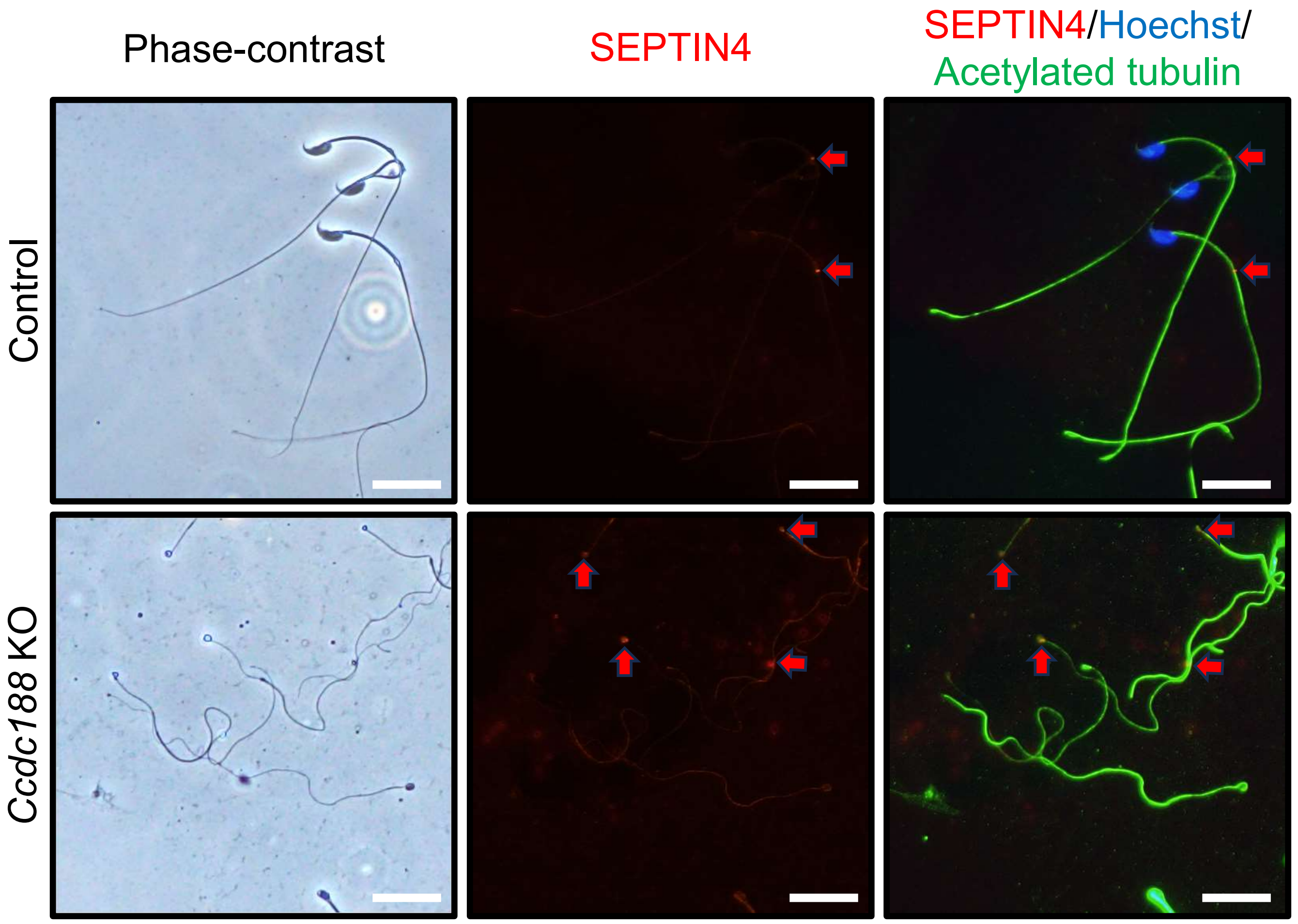

Figure S5. *Ccdc188*-null spermatozoa lack a midpiece
Immunofluorescent analysis of control and *Ccdc188* KO spermatozoa by SEPTIN4 (red), acetylated tubulin (green), and Hoechst 33342 (blue) to visualize the annulus, microtubules, and nuclei, respectively. The SEPTIN4 signal (red arrows) appeared to be observed at the border between the midpiece and the principal piece of the control spermatozoa, while it was not observed or observed at the edge of the *Ccdc188* KO flagellum. Scale bars: 20 μm.
