# Supplementary material for: Loss of CCDC188 causes male infertility with defects in the sperm head-neck connection in mice
Source: Biol Reprod. Author manuscript; Available in PMC 2025 Jan 17. (PMC11736427; doi:10.1093/biolre/ioae137)
Supplement: Supplementary tables [file NIHMS2032238-supplement-Supplementary_tables.pdf]

Purpose	Primer sequence	Annealing temperature (°C)	Cycles
mouse *Ccdc188* RT-PCR	Fw: CAGACAAGAAGGGGAGGCTG<br>Rv: TCTGTTGCAGCTCACTCTGG	60	35
mouse *Actb* RT-PCR	Fw: CATCCGTAAAGACCTCTATGCCAAC<br>Rv: ATGGAGCCACCGATCCACA	60	35
Ccdc188 WT allele genotyping	Fw: ATGGCCATTACTGAGGTGCC<br>Rv: ACATAGCTTCAGGACCGTGC	60	40
Ccdc188 KO allele genotyping	Fw: AATGATCTTGGCCGGACACT<br>Rv: TAGGCAAGGACCAGTAAGGC	60	40

Table S1. Primer list

Immunofluorescence analysis

Antibody	Clone No.	Host species	Catalog No.	Company	Dilution for testis	Dilution for sperm
anti-acetylated tubulin	6-11B-1	Mouse	ab24610	abcam	1:500	1:500
anti-Septin4	polyclonal	Rabbit	18987	IBL	-	1:100
anti-TOMM20	EPR15581-54	Rabbit	ab186735	abcam	-	1:100

Table S2. Antibody list
